# Willingness to Receive a COVID-19 Vaccination Among Incarcerated or Detained Persons in Correctional and Detention Facilities — Four States, September–December 2020

**DOI:** 10.15585/mmwr.mm7013a3

**Published:** 2021-04-02

**Authors:** Marc F. Stern, Alexandra M. Piasecki, Lara B. Strick, Poornima Rajeshwar, Erika Tyagi, Sharon Dolovich, Priti R. Patel, Rena Fukunaga, Nathan W. Furukawa

**Affiliations:** ^1^University of Washington, Seattle; ^2^CDC COVID-19 Response Team; ^3^Washington State Department of Corrections; ^4^University of California, Los Angeles.

Incarcerated and detained persons are at increased risk for acquiring COVID-19. However, little is known about their willingness to receive a COVID-19 vaccination. During September–December 2020, residents in three prisons and 13 jails in four states were surveyed regarding their willingness to receive a COVID-19 vaccination and their reasons for COVID-19 vaccination hesitancy or refusal. Among 5,110 participants, 2,294 (44.9%) said they would receive a COVID-19 vaccination, 498 (9.8%) said they would hesitate to receive it, and 2,318 (45.4%) said they would refuse to receive it. Willingness to receive a COVID-19 vaccination was lowest among Black/African American (Black) (36.7%; 510 of 1,390) persons, participants aged 18–29 years (38.5%; 583 of 1,516), and those who lived in jails versus prisons (43.7%; 1,850 of 4,232). Common reasons reported for COVID-19 vaccine hesitancy were waiting for more information (54.8%) and efficacy or safety concerns (31.0%). The most common reason for COVID-19 vaccination refusal was distrust of health care, correctional, or government personnel or institutions (20.1%). Public health interventions to improve vaccine confidence and trust are needed to increase vaccination acceptance by incarcerated or detained persons.

Correctional and detention facilities are at increased risk for COVID-19 outbreaks because of several factors, including difficulty maintaining physical distancing, limited space for isolation or quarantine, poor ventilation, and limited resources for testing and personal protective equipment (*1*). Members of racial and ethnic minority groups and persons with unstable housing, substance use disorders, and mental illness are disproportionately incarcerated and more likely to have chronic diseases resulting from disparities in social determinants of health (*2*). The higher prevalence of medical conditions associated with severe COVID-19 illness, delays in timely diagnosis, and inadequate treatment might all contribute to the increased risk for death from COVID-19 among incarcerated or detained persons (*3*). Recognizing this, some jurisdictions have prioritized incarcerated or detained persons for COVID-19 vaccination.* Data pertaining to willingness of residents to be vaccinated while incarcerated or detained are scarce, and routine vaccination coverage is low in these settings (*4*).

To assess attitudes related to receipt of COVID-19 vaccination, a survey was conducted in three prisons and 10 small-to-medium–sized jails in Washington and one medium-to-large–sized jail each in California, Florida, and Texas, which agreed to participate in an evaluation of willingness of their residents to receive a COVID-19 vaccination.^†^ The survey was conducted during September 22–December 12, 2020, with 96% of the interviews occurring during November 20–December 12. Interviews were conducted by health care providers, health service administrators, or corrections staff members, depending on the facility and staff member availability. Interviewers attempted to interview all residents within a facility, except those who were in special housing units, quarantine, or medical isolation. Interviews were conducted one-on-one or in small groups in outdoor spaces, communal lounges, or individual cells. Participation was voluntary, and participants could stop the interview at any time. The survey participation rate was 64.2% (range = 20.0%–94.0%).^§^

Interviewers informed participants that correctional and detention facilities were planning for COVID-19 vaccination. Participants were asked “When a vaccine for COVID-19 is approved, will you sign-up to get it?” (willingness). Possible responses were “yes,” “no,” or “maybe.” Participants who answered “no” (refusal) or “maybe” (hesitancy) were then asked to provide a reason for vaccine refusal or hesitancy; responses were open-ended. The primary reason reported for vaccination hesitancy or refusal was coded into one of seven categories: 1) having concerns about efficacy or safety; 2) awaiting more information or to see others vaccinated; 3) distrust of health care, correctional, or governmental personnel or institutions; 4) not perceiving themselves at risk for COVID-19 or perceiving vaccination as unnecessary; 5) being against vaccination in general; 6) believing in a virus- or vaccine-related conspiracy to harm incarcerated or detained persons; or 7) other reasons.

Willingness to receive a COVID-19 vaccination was compared by incarcerated or detained persons’ sex, age, and race/ethnicity and facility type and location. Categories of reasons for vaccine refusal or hesitancy were compared by willingness to receive a vaccination. Multivariable logistic regression was used to compare willingness to receive a vaccination (“yes”) and vaccine hesitancy or refusal (“maybe”/“no”) by demographic characteristics, facility type, and location. Analyses were conducted using SAS (version 9.4; SAS Institute) and R software (version 4.0.3; the R Foundation). The University of Washington Institutional Review Board determined that this was an evaluation to inform educational needs and not human subjects research. This activity was reviewed by CDC and was conducted consistent with applicable federal law and CDC policy.^¶^

Among 5,110 total participants, a total of 2,294 (44.9%) responded that they would receive, 498 (9.7%) that they would hesitate to receive, and 2,318 (45.4%) that they would refuse a COVID-19 vaccination (Table 1). Willingness to receive a vaccination was similar among men (45.0%) and women (44.4%), but a higher percentage of women than men indicated they would hesitate to receive a COVID-19 vaccination (14.9% versus 8.7%; p<0.001 for “yes” versus “maybe” by sex). Willingness to receive a COVID-19 vaccination increased with age from 38.5% among participants aged 18–29 years to 61.8% among those aged 60–83 years (p<0.001). Willingness to receive a vaccination was lowest among Black participants (36.7%) and highest among Hispanic/Latino (Hispanic) (52.5%) and American Indian/Alaska Native (48.4%) participants (p<0.001 for group). Willingness to receive a COVID-19 vaccination was higher among participants in prisons than among those in jails (50.6% versus 43.7%; p<0.001). Lower willingness to receive a vaccination persisted in multivariable analyses among participants who were younger, were Black, lived in jails, and were not in California. When stratified by sex and race/ethnicity, willingness to receive a vaccination was highest among Hispanic men (54.9%) and lowest among Hispanic women (35.8%) and Black men (36.0%) (Table 2).

**TABLE 1:** Willingness to receive a COVID-19 vaccination among incarcerated or detained persons, by demographics and facility — four states, September–December 2020

Characteristic (no. with available information)	Willingness to receive a COVID-19 vaccination when it is authorized, no. of persons (%)				Willingness to be vaccinated,* odds ratio (95% CI)	
	All persons	Yes	Maybe	No	Unadjusted^†^	Adjusted^§^
**Total no. of persons**	**5,110 (100)**	**2,294 (44.9)**	**498 (9.7)**	**2,318 (45.4)**	—	—
**Sex^¶^ (5,107)**						
Men	4,209 (82.4)	1,895 (45.0)	364 (8.7)	1,950 (46.3)	Referent	Referent
Women	898 (17.6)	399 (44.4)	134 (14.9)	365 (40.7)	0.98 (0.85–1.13)	1.04 (0.86–1.24)
**Age group, yrs^¶^ (5,070)**						
18–29	1,516 (29.9)	583 (38.5)	136 (9.0)	797 (52.6)	Referent	Referent
30–39	1,589 (31.3)	653 (41.1)	181 (11.4)	755 (47.5)	1.12 (0.97–1.29)	1.09 (0.94–1.26)
40–49	1,011 (19.9)	496 (49.1)	111 (11.0)	404 (40.0)	1.54 (1.31–1.81)	1.46 (1.24–1.73)
50–59	661 (13.0)	367 (55.5)	51 (7.7)	243 (36.8)	2.00 (1.66–2.40)	1.92 (1.58–2.32)
60–83	293 (5.8)	181 (61.8)	18 (6.1)	94 (32.1)	2.59 (1.99–3.35)	2.62 (2.01–3.43)
**Race/Ethnicity^¶^ (4,979)**						
White	2,085 (41.9)	915 (43.9)	251 (12.0)	919 (44.1)	Referent	Referent
Black/African American	1,390 (28.0)	510 (36.7)	105 (7.6)	775 (55.8)	0.74 (0.65–0.85)	0.74 (0.63–0.86)
Hispanic/Latino	1,013 (20.4)	532 (52.5)	75 (7.4)	406 (40.1)	1.41 (1.22–1.64)	1.34 (1.14–1.58)
American Indian/Alaska Native	221 (4.4)	107 (48.4)	19 (8.6)	95 (43.0)	1.20 (0.91–1.58)	1.36 (1.02–1.80)
Other	270 (5.4)	128 (47.4)	43 (15.9)	99 (36.7)	1.15 (0.89–1.49)	0.93 (0.71–1.21)
Facility type^¶,^** (5,110)						
Jail	4,232 (82.8)	1,850 (43.7)	345 (8.2)	2, 037 (48.1)	Referent	Referent
Prison	878 (17.2)	444 (50.6)	153 (17.4)	281 (32.0)	1.32 (1.14–1.52)	1.23 (1.01–1.51)
**Facility location^¶^ (5,110)**						
Washington	2,514 (49.2)	1,081 (43.0)	349 (13.9)	1,084 (43.1)	Referent	Referent
Florida	2,015 (39.4)	856 (42.5)	67 (3.3)	1,092 (54.2)	0.98 (0.87–1.10)	1.17 (0.99–1.36)
California	449 (8.8)	297 (66.2)	35 (7.8)	117 (26.1)	2.59 (2.10–3.20)	2.96 (2.35–3.72)
Texas	132 (2.6)	60 (45.5)	47 (35.6)	25 (18.9)	1.11 (0.78–1.57)	1.41 (0.98–2.04)

**Abbreviation:** CI = confidence interval.

* Bivariate and multivariable regression compared willingness to receive a vaccination (“yes”) and vaccine hesitancy or refusal (“maybe”/“no”).

^†^ Separate bivariate logistic regression models were constructed to examine the association between participant’s willingness to receive a COVID-19 vaccination by sex, age, race/ethnicity, facility type, and location.

^§^ A multivariable logistic regression model was constructed to examine the association between participant’s willingness to receive a COVID-19 vaccination, by sex, age, race/ethnicity, facility type, and location, adjusting for all characteristics listed in the table.

^¶^ p<0.001 comparing “yes,” “maybe,” and “no” vaccination willingness and characteristic (by chi-square test).

** Prisons usually hold persons with sentences >1 year and jails hold persons pretrial or with sentences ≤1 year.

**TABLE 2:** Willingness to receive a COVID-19 vaccination among incarcerated or detained persons, by sex and race/ethnicity — four states,* September–December 2020

Race/Ethnicity	All persons	Willingness to receive a COVID-19 vaccination when it is authorized, no. of persons (%)					
		Men^†^ (n = 4,081)			Women^§^ (n = 894)		
		Yes	Maybe	No	Yes	Maybe	No
**Total**	**4,975 (100)**	**1,795 (44.0)**	**359 (8.8)**	**1,927 (47.2)**	**396 (44.3)**	**134 (15.0)**	**364 (40.7)**
White	2,084 (41.9)	687 (43.4)	172 (10.1)	724 (45.7)	227 (45.3)	79 (15.8)	195 (38.9)
Black/African American	1,390 (27.9)	453 (36.0)	89 (7.1)	717 (57.0)	57 (43.5)	16 (12.2)	58 (44.3)
Hispanic/Latino	1,011 (20.3)	489 (54.9)	54 (6.1)	348 (39.1)	43 (35.8)	21 (17.5)	56 (46.7)
American Indian/Alaska Native	221 (4.4)	64 (46.0)	11 (7.9)	64 (46.0)	43 (52.4)	8 (9.8)	31 (37.8)
Other	269 (5.4)	102 (48.1)	33 (15.8)	74 (35.4)	26 (43.3)	10 (16.7)	24 (40.0)

* California, Florida, Texas, and Washington.

^†^ p<0.001 comparing “yes,” “maybe,” and “no” vaccination willingness and race/ethnicity among men (by chi-square test).

^§^ p<0.001 comparing “yes,” “maybe,” and “no” vaccination willingness and race/ethnicity among women (by chi-square test).

Among 2,816 participants who indicated they would hesitate to receive or would refuse a COVID-19 vaccination, 2,281 (81.0%) provided a primary reason. Among 458 participants who would hesitate to receive a COVID-19 vaccination, 251 (54.8%) said they were awaiting more information or to see others vaccinated, and 142 (31.0%) had concerns about efficacy or safety (Table 3). Among 1,823 participants who would refuse a COVID-19 vaccination, 366 (20.1%) cited distrust of health care, correctional, or governmental personnel or institutions; 358 (19.6%) had concerns about efficacy or safety; and 344 (18.9%) did not perceive themselves to be at risk for COVID-19 or thought vaccination was unnecessary.

**TABLE 3:** Primary reasons for COVID-19 vaccination hesitancy or refusal among incarcerated or detained persons, by vaccination willingness — four states,* September–December 2020

Primary reason for refusal or hesitancy^†^	Willingness to receive a COVID-19 vaccination when it is authorized,^§^ no. of persons (%)		
	All respondents (N = 2,281)	Maybe (hesitancy) (n = 458)	No (refusal) (n = 1,823)
Efficacy or safety concerns	500 (21.9)	142 (31.0)	358 (19.6)
Awaiting more information or to see others vaccinated	493 (21.6)	251 (54.8)	242 (13.3)
Distrust of health care, correctional, or governmental personnel or institutions	379 (16.6)	13 (2.8)	366 (20.1)
Not perceiving themselves at risk for COVID-19 or perceiving vaccination as unnecessary	353 (15.5)	9 (2.0)	344 (18.9)
Against vaccination in general	253 (11.1)	5 (1.1)	248 (13.6)
Believing in a virus- or vaccine-related conspiracy to harm incarcerated or detained persons	104 (4.6)	4 (0.9)	100 (5.5)
Other	199 (8.7)	34 (7.4)	165 (9.1)

* California, Florida, Texas, and Washington.

^†^ Participants were then asked to provide a reason for vaccination refusal or hesitancy with open-ended responses that were coded into one of seven primary reasons.

^§^ p<0.001 comparing the distribution of reasons between “maybe” and “no” vaccination willingness (by chi-square test).

## Discussion

Among incarcerated or detained persons, willingness to receive a COVID-19 vaccination was lower in this analysis compared with findings from national surveys of the general population over the same period (45% versus 56%–67%) (*5*,*6*). This survey identified significantly lower prevalences of willingness to receive a vaccination among persons who were younger or Black, consistent with similar surveys in the general population. Lower willingness to receive a COVID-19 vaccination among Black participants is not unexpected given historical mistreatment and higher rates of distrust of health care, correctional, and governmental institutions** (*7*). This finding is particularly concerning because this group is at higher risk for severe illness and death from COVID-19 and overrepresented in the criminal justice system (*8*).

More than three fourths of participants who reported they were hesitant to receive a COVID-19 vaccination indicated that they had concerns about efficacy or safety or were waiting to see others vaccinated. This finding highlights the need for access to COVID-19 vaccination information that is culturally relevant and appropriate for persons of all health literacy levels, and that is conveyed via multiple formats and languages including video messages, handouts, posters, presentations, peer interactions, and discussions with experts. In addition, opportunities to observe peers, family members, and trusted influencers having positive vaccination experiences could increase vaccine confidence. Nearly one in five participants who would refuse vaccination did not perceive themselves to be at risk for COVID-19 and thought vaccination was unnecessary. Interventions to increase COVID-19 risk perception and highlight the facility and community protective benefits of vaccination might further increase vaccination acceptance. Participants who would refuse vaccination distrusted service providers or the government (20%), were against vaccination in general (14%), or believed a COVID-19 vaccination was a conspiracy to harm them (6%). These reasons were much more prevalent among incarcerated or detained persons than the prevalence that has been reported among the general population (*7*), suggesting that overcoming systemic distrust will be necessary to increase willingness to receive a vaccination among a segment of this population. Interventions to improve vaccine confidence should not be punitive or overly generous to avoid being perceived as coercive, which might worsen trust.

The findings in this report are subject to at least three limitations. First, the correctional and detention facilities were selected based on voluntary participation and are likely not representative of facilities or their residents nationwide. Second, the survey was largely conducted before Emergency Use Authorization of the first COVID-19 vaccine on December 11, 2020; willingness to be vaccinated might have changed given the rapid evolution of the pandemic and vaccine rollout. Finally, stated willingness to receive a COVID-19 vaccination is an imperfect measure of whether a person will accept a vaccination when it is actually offered (*9*). Persons might be influenced by a variety of peer, environmental, and situational factors that support or hinder receipt of vaccination.

This report underscores the urgent need for interventions that are culturally relevant and appropriate for various health literacy levels to increase vaccine confidence among incarcerated or detained persons. Incarcerated or detained persons might have inherent higher distrust of governmental systems based on their interactions with law enforcement or the justice system or their experiences with institutional racism, emphasizing the need for trusted messengers to directly appeal to these persons. Low vaccine confidence contributes to low vaccination coverage and risks further exacerbating preexisting inequities in COVID-19 outcomes by incarceration or detention status, age, and race/ethnicity. In addition, correctional or detention facilities staff members and residents frequently cycle between the facility and the community. COVID-19 outbreaks in these settings can contribute to community transmission, which intensifies the importance of preventing outbreaks in this setting with vaccination (*10*). It is critically important for public health and criminal justice professionals to promote health equity by addressing vaccine hesitancy and improving vaccine confidence and acceptance among this disproportionately affected population.

**Summary**

**What is already known about this topic?**

Persons living in correctional or detention facilities are at increased risk for COVID-19. Certain jurisdictions have prioritized COVID-19 vaccination of incarcerated or detained persons.

**What is added by this report?**

Among incarcerated or detained participants at correctional and detention facilities in four states who were surveyed before authorization of COVID-19 vaccines for emergency use, 45% were willing to be vaccinated. Willingness to be vaccinated was lower among participants who were younger, identified as Black/African American, and lived in jails.

**What are the implications for public health practice?**

COVID-19 outbreaks among incarcerated or detained persons can exacerbate inequities in COVID-19 outcomes and contribute to community transmission. Interventions are needed to improve vaccine confidence among incarcerated or detained persons.

## Footnotes

* Some jurisdictions have specifically prioritized staff members, residents of correctional or detention facilities, or both in their COVID-19 vaccination plans. https://www.cdc.gov/vaccines/covid-19/covid19-vaccination-guidance.html

† Prisons usually hold persons with sentences >1 year and jails hold persons pretrial or with sentences ≤1 year.

§ 45 C.F.R. part 46, 21 C.F.R. part 56; 42 U.S.C. Sect. 241(d); 5 U.S.C. Sect. 552a; 44 U.S.C. Sect. 3501 et seq.

¶ The denominator for the response rate was resident census of each facility, which included persons in restricted areas who were not approached for interview. Because not all facility residents were approached, the response rate was at least 64.2% (5,110 participants among a cumulative census of 7,955).

** Long-standing systemic health and social inequities have put many persons from racial and ethnic minority groups at increased risk for becoming ill and dying from COVID-19. Inequities in the social determinants of health have historically prevented these groups from having the same opportunities for economic, physical, and emotional health. These inequities are highlighted by the factors that contribute to increased risk for COVID-19 exposure, severe illness from COVID-19, death, and unintended consequences of COVID-19 mitigation strategies. https://www.cdc.gov/coronavirus/2019-ncov/community/health-equity/racial-ethnic-disparities/index.html
