# *Erratum:* Vol. 70, No. 13

**DOI:** 10.15585/mmwr.mm7015a6

**Published:** 2021-04-16

In the report “Willingness to Receive a COVID-19 Vaccination Among Incarcerated or Detained Persons in Correctional and Detention Facilities — Four States, September–December 2020,” on page 473, the third footnote should have read, “**^§^ The denominator for the response rate was resident census of each facility, which included persons in restricted areas who were not approached for interview. Because not all facility residents were approached, the response rate was at least 64.2% (5,110 participants among a cumulative census of 7,955).**” On page 474, the footnote should have read, “**^¶^ 45 C.F.R. part 46, 21 C.F.R. part 56; 42 U.S.C. Sect. 241(d); 5 U.S.C. Sect. 552a; 44 U.S.C. Sect. 3501 et seq.**”

